# Physical activity in out of school hours care: an observational study

**DOI:** 10.1186/s12966-021-01197-6

**Published:** 2021-09-16

**Authors:** Ruth K. Crowe, Yasmine C. Probst, Rebecca M. Stanley, Sarah T. Ryan, R. Glenn Weaver, Michael W. Beets, Jennifer A. Norman, Susan E. Furber, Cecilia Vuong, Megan L. Hammersley, Karen Wardle, Lisa Franco, Marc Davies, Christine Innes-Hughes, Anthony D. Okely

**Affiliations:** 1 Early Start, Building 21, Ring Road, Keiraville, NSW 2522 Australia; 2 School of Medicine, Faculty of Science Medicine and Health, University of Wollongong, Northfields Avenue, NSW Wollongong, Australia; 3 Illawarra Health and Medical Research Institute, University of Wollongong, Wollongong, NSW Australia; 4 Early Start, Faculty of the Arts Social Sciences and Humanities, University of Wollongong, Wollongong, NSW Australia; 5 Exercise Science, University of South Carolina, Columbia, SC USA; 6 Health Promotion Service, Illawarra Shoalhaven Local Health District, Warrawong, NSW Australia; 7 Health Promotion Service, South Western Sydney Local Health District, Liverpool, NSW Australia; 8 Centre for Population Health, St Leonards, NSW Australia

**Keywords:** Physical activity, Physical activity environment, MVPA, Out of school hours care, Afterschool care, Child care, Primary-school children

## Abstract

**Background:**

Opportunities for physical activity within out of school hours care (OSHC) are not well documented in Australia. This study explored factors associated with children (5–12 years) meeting 30 min of moderate to vigorous physical activity (MVPA) while attending OSHC in the afternoon period.

**Methods:**

A cross-sectional study, conducted in 89 OSHC services in New South Wales, Australia, serving 4,408 children. Each service was visited twice between 2018–2019. Physical activity promotion practices were captured via short interviews and System for Observing Staff Promotion of Physical Activity and Nutrition (SOSPAN). Physical activity spaces was measured (m^2^) and physical activity of 3,614 child days (42% girls), were collected using Acti-Graph accelerometers. Association between program practices and children accumulation of MVPA was tested using mixed effects logistic regression, adjusted by OSHC service and child.

**Results:**

Twenty-six percent of children (n = 925) accumulated 30 min or more of MVPA. Factors associated with children reaching MVPA recommendations included: services scheduling greater amounts of child-led free play, both 30–59 min (OR 2.6, 95%CI 1.70, 3.98) and ≥ 60 min (OR 6.4, 95%CI 3.90, 10.49); opportunities for staff-led organised play of ≥ 30 min (OR 2.3, 95%CI 1.47, 3.83); and active games that engaged the majority of children (OR 1.7, 95%CI 1.11, 2.61). Children were less likely to meet MVPA recommendations if services played games with elimination components (OR 0.56, 95%CI 0.37, 0.86).

**Conclusion:**

Improvements to service-level physical activity promotion practices, specifically the type of physical activity scheduled and the structure of games, may be an effective strategy to increase MVPA of children attending OSHC afterschool in NSW, Australia.

## Introduction

Moderate-to-vigorous physical activity (MVPA) is a vital part of a healthy lifestyle. Regular engagement in MVPA during childhood has not only been associated with numerous physical health outcomes including the protection against non-communicable diseases (e.g. type 2 diabetes mellitus, cardiovascular disease), and disease risk factors (e.g. high blood pressure and high cholesterol, overweight and obesity), [1] it is also associated with positive social and emotional health implications (e.g. reduced depression and anxiety) [2]. Like many countries, Australia recommends children accumulate a minimum of 60 min of MVPA each day [3], however, only 1 in 4 Australian children (aged 5-12 years) are meeting this recommendation [3]. 

With the vast majority of a child’s school day spent sedentary, the afterschool period (15:00 -18:00 h) has been identified as a key time for children to accumulate up to half (30 min) of their recommended daily MVPA [4–6]. Out of school hours care (OSHC), under which sits afterschool care, is the second largest childcare setting in Australia, with more than 450,000 children attending each year [7]. However, device-measured physical activity (accelerometry) within OSHC is not well documented in Australia. Therefore, this study aimed to a) describe the physical activity environments in OSHC; b) explore factors associated with children meeting 30 min of MVPA while attending OSHC in the afterschool time period; and c) determine MVPA levels of children while attending OSHC.

## Methods

### Participants and setting

A total of 243 OSHC services were registered on the Australian Children’s Education and Care Quality Authority (ACECQA) website, in 2017, at the time recruitment commenced. OSHC services were eligible to participate in the study if they a) provided care to primary-school aged children (Kindergarten to 6^th^ grade) b) operated during afterschool hours (15:00 – 18:00 h), c) were located within the Illawarra Shoalhaven or South Western Sydney Local Health Districts in New South Wales, Australia, and d) had more than five students enrolled per day. Once the eligibility criteria were applied, a total of 161 OSHC services were contacted for recruitment. Written informed consent was obtained by each OSHC director. All parents/ guardians had the ability to opt-out (passive consent) their children from the research at any time. The study was advertised to staff, parents and guardians, for a minimum of two weeks prior to the study commencement and throughout the data collection period. Advertisement consisted of several methods; including a) recruitment video and electronic participant information sheets/ opt-out forms were disseminated by each respective OSHC service to all families and employees; b) research notification posters, were displayed at each OSHC entrance, sign in/ out desks and on notice boards; and c) participant information sheets and opt-out forms were available at each sign in/out areas. All children were invited to wear an accelerometer while in attendance at the OSHC, unless parents/ guardians had opted their child out. All children were given the option to refuse their assent on the day of data collection [6, 8]. Ethical approval was granted by the University of Wollongong Human Research Ethics Committee (HE17/490). A brief description of the study procedures are provided below and a detailed protocol has been published elsewhere [8].

### Physical Activity Measurements

Child physical activity was objectively measured using Acti-Graph GT3X + model( ActiGraph Corporation, Pensacola, FL) accelerometers, initialised at a sampling rate of 30Hertz. Upon arrival at the OSHC, children were fitted with an accelerometer, worn around the waist sitting at the right-hip. The time accelerometers were fitted was recorded (time-on) and child demographics (school grade and sex) were also collected. As children departed the service, data collectors, stationed near the exit, removed the device and recorded the time (time-off). A valid day of accelerometry data was defined as a child wearing the accelerometer for at least 60 min while in attendance at a service [9, 10].

### Physical activity policies and practices

Physical activity policies and practices were initially captured via short, structured interviews with the service directors. The interview questions were guided by the validated Healthy Afterschool Activity and Nutrition Document (HAAND) tool [11] and captured information on service policies and practices, including: a) the presence of a physical activity policy, b) staff training, c) the use of physical activity promotion materials, d) the inclusion of children’s voices when planning daily programs (children’s feedback), and e) the use of recreational screen-time (TV and handheld devices e.g. tablets/ smart phones).

The System for Observing Staff Promotion of Physical Activity and Nutrition (SOSPAN) [12] was used to capture physical activity promotion practices and behaviours, including the type and structure of physical activity opportunities within OSHC programs [12, 13]. In brief, each service was visited, unannounced, on two non-consecutive days by trained data collectors between March 2018 – April 2019. The data were captured via continuously scanning, from left to right, in all rooms and zones consisting of five or more children and at least one staff member. Data collectors systematically rotated between zones, performing a minimum of five scans in each zone before moving into a different area. Scans were continuously completed from the commencement of the session until the end of the program or until less than five children remained at the service. Physical activity behaviours were coded as either: a) free play, which consisted of children playing in an unstructured manner with no direction or input from adults/ staff, b) organised play, usually involving structured games or activities with rules directed by staff (e.g. softball, dodgeball or stuck in the mud), and c) enrichment, a non-physical activity (e.g. reading, craft, quiet-play, or homework) typically performed indoors. Other behaviours captured, included: a) the level of staff interaction ( i.e. supervising only, engaging in physical activity, encouraging physical activity  or leading/ instructing an activity), b) if games were able to engage a majority of the children, c) stand and wait time (i.e. were children lining-up and waiting their turn to play in a game), or d) elimination games (i.e. were participants eliminated from a game when they were deemed “out”).

### Available physical activity space

Indoor and outdoor spaces, accessible to children, were identified by staff prior to data collection. Designated areas were divided into zones and identified as physical activity areas (eg. open fields, basketball courts, fixed-equipment) or non-physical activity areas (classrooms, halls) and measured (metre^2^) using a Craftright measuring wheel by data collectors.

### Observer training and reliability

Data collectors were trained over a three-day period, using a combination of classroom simulations and field practice prior to the study commencement. Data collectors were required to meet > 80% interrater-reliability via an interval-by-interval agreement on two consecutive days prior to data collection. Reliability scans were collected on each data collection day, with a minimum of 30% of scans used to calculate reliability [14]. Interrater-reliability was calculated using percentage agreement and Cohen’s Kappa [15]. The median percentage agreement was 91% and a Kappa coefficient of 0.97 (ranging from 0.81 to 1.00).

### Statistical analysis

All accelerometry data was downloaded in 15-s epochs from ActiLife software and physical activity levels were calculated in Python software using Evenson cut-points [16, 17]. All descriptive means, standard deviations, frequencies, percentages and independent t-tests were calculated using SPSS software (v26, IBM Corporation, Armonk, NY, USA). All physical activity practices and behaviours were coded dichotomously, as either observed/reported or not observed/reported. The accumulated time (minutes) spent in various activity types (free-play, organised play and screen-time) were calculated using Excel software and the predefined activity categories and time stamping captured within the SOSPAN instrument. The association between program practices and children meeting 30 min of MVPA was tested using mixed effects logistic regression, adjusted by OSHC service and child. The mixed effects logistic regression was conducted using STATA software (V. 15.1, College Station, TX). Statistical analysis was completed in February 2021.

## Results

Of the 161 eligible OSHC services contacted, 89 participated (55%). Seventy-four (83%) services were located on school grounds, nine (10%) in early childhood settings, five (6%) in community centres and one (1%) in a faith-based location. On average, sessions ran for 180 (± 16.8) minutes and provided opportunities for children to be physically active for 97 (± 41.1) minutes of the session. A total of 4,408 children attended the services, with 3,614 children wearing an accelerometer for a minimum of 60 min on at least one observation day. Children spent an average of 70.6 (± 23.5) minutes in total physical activity and 22 (± 12.8) minutes in MVPA/day (Table 1). Boys spent significantly more time in MVPA and less time sedentary (*p* < 0.001) than girls.

**Table 1:** Sedentary and physical activity levels of children attending Out of School Hours Care (OSHC) services in the afterschool period

						***Independent t-test***	
Physical activity levels	**Minutes** **Mean (SD)**	**Minutes** **Mean (SD)**	**Std. Err**	**Minutes** **Mean (SD)**	**Std. Err**	**Sig**	**95% CI**
***Mean time (minutes) spent sedentary and in physical activity levels***							
	**All children (n = 3614)**	**Girls (n = 1521)**		**Boys (n = 1514)**			
*Sedentary*	36.0 (19.5)	41.1 (19.8)	0.51	31.1 (18.0)	0.46	** < .001**	-11.38, -8.68
*Light physical activity*	48.4 (16.3)	48.5 (16.2)	0.42	49.3 (16.7)	0.43	.148	-0.31, 2.04
*Moderate-to-vigorous physical activity*	22.1 (12.8)	18.5 (10.9)	0.28	25.7 (13.6)	0.35	** < .001**	6.38, 8.14
*Total physical activity*	70.6 (23.5)	67.0 (22.5)	0.58	75.0 (24.0)	0.62		
		**Grade K—2 (n = 1709)**		**Grade 3—6 (n = 1326)**			
*Sedentary*		33.9 (18.6)	0.45	38.8 (20.5)	0.56	**.001**	-6.33, -3.54
*Light physical activity*		49.6 (18.0)	0.43	46.2 (16.5)	0.45	** < .001**	2.86, 5.20
*Moderate-to-vigorous physical activity*		22.4 (12.8)	0.31	21.7 (12.7)	0.35	.167	-0.27, 1.57
*Total physical activity*		73.2 (23.6)	0.57	68.4 (23.4)	0.64		
***Mean percentage (%) of wear time spent sedentary and in physical activity levels***							
	**All children (n = 3614)**	**Girls (n = 1521)**		**Boys (n = 1514)**			
*Sedentary*	33.5 (15.5)	37.7 (15.1)	0.39	29.1 (14.5)	0.37	** < .001**	-9.76, -7.65
*Light physical activity*	45.5 (10.3)	45.0 (10.4)	0.27	46.4 (10.2)	0.26	** < .001**	0.75, 2.22
*Moderate-to-vigorous physical activity*	21.0 (11.4)	17.2 (9.5)	0.25	24.5 (11.8)	0.30	** < .001**	6.50, 8.00
*Total physical activity*	66.5 (15.5)	62.2 (15.1)	0.38	70.9 (14.5)	0.37		
		**Infant (n = 1709)**		**Primary (n = 1326)**			
*Sedentary*		31.5 (15.0)	0.36	35.9 (15.7)	0.43	** < .001**	-5.54, -3.35
*Light physical activity*		47.3 (10.1)	0.24	43.5 (10.2)	0.28	** < .001**	3.05, 4.50
*Moderate-to-vigorous physical activity*		21.1 (11.4)	0.28	20.5 (11.2)	0.31	.109	-0.15, 1.48
*Total physical activity*		68.5 (14.9)	0.36	64.1 (15.7)	0.43		

Note: Missing descriptive (sex, age group) data of 579 children

Bolded values are significant P < 0.05

K—2: kindergarten to year 2; Grade 3—6: years 3 – 6

A total of 9,218 SOSPAN scans were completed across 178 observation days. The percentage of time spent in different activities comprised: 52% physical activity (38% child-led free play and 15% staff-led organised play), 43% enrichment, and 4% afternoon snack. Staff supervised children 98% of the time, engaged in physical activity with children 9% of the time, and instructed or led physical activities 11% of the time. A total of 26% of children met the criterion of 30 min or more of MVPA. Children who attended services that provided a combination of child-led free play with staff-led organised play spent significantly more time in MVPA than those services which only offered free play or organised play opportunities (Table 2).

**Table 2:** Characteristics of reported and observed practices, environments and accumulated child physical activity across Out of School Hours Care (OSHC) services

Reported Program Practices (Short interview)		Freq	%	Mean (SD)
Written physical activity policy		20	22	
Physical activity promotion training by a certified training		26	29	
Physical activity promotion material used		18	20	
Children’s voices and preferences are included in daily programming		87	97	
^**b**^**Available Physical Activity Space (m**^**2**^**)**				
Outdoor				3,616.6 (3182.7)
Indoor				70.3 (98.3)
**Observed Physical Activity Practices**		**178**	**100**	
Child-led free play				
	< 29 min	40	22	
	30-59 min	54	30	
	> 60 min	83	46	
Staff-led organised play				
	None	98	55	
	< 29 min	34	19	
	> 30 min	41	23	
Recreational screen-time		55	30	
	> 30 min	34	19	
Recreational handheld devices (i-pads, tablets, smart-phones)		54	30	
Active games that engage the majority of children		46	25	
Active games consisting of elimination components		46	25	
Child stand and wait games		15	8	
Staff engage in physical play		104	58	
Homework		45	25	
^**d**^**Mean minutes of moderate-to-vigorous physical activity (MVPA) accumulated in different activities types**				
Child-led free play only		1869	52	21.5 (12.8)
Staff-led organised play		119	3	16.3 (10.5)
Both free play and organised play		1573	44	**24.0 (12.6)**
None		52	1	8.2 (8.0)
**% of children meeting 30 min > MVPA**				**Pearson’s Chi Square**
**Total children**		**925**	**26**	
^a^Boys (n = 1514)		546	15	***p* < .001**
^a^Girls (n = 15,521)		235	6.5	
^a^**Grade** K—Year 2 (n = 1709)		453	12	*p .366*
^a^**Grade** 3 – Year 6 (n = 1326)		327	9	

Note: Bolded values are significant P < 0.05

^**a**^Missing descriptive (sex, age) data of 579 children

^b^Measured on site, in metres using a craft.right measuring wheel

^c^Physical activity data collected using Actigraph wGT3X-BT model accelerometers

Services that provided opportunities for 30–59 min (OR = 2.6; 95%CI 1.7–4.0), or more than 60 min of child-led free play (OR = 6.4; 95%CI 3.9–10.5); 30 min or more of scheduled staff-led organised play (OR = 2.3; 95%CI 1.5–3.8); or facilitated games which engaged the majority of children (OR = 1.7; 95%CI 1.1–2.6), were more likely to have children meet 30 min of MVPA while attending OSHC services (Table 3). Services that played games with children that included an elimination component were less likely to meet the recommended 30 min of MVPA than those that did not (OR = 0.6; 95%CI 0.4–0.9). The presence of a physical activity policy, staff training, the use of physical activity promotion material, recreational screen-time, incorporating child activity preferences, stand and wait games and staff engaging in physical activity with children were not associated with children meeting 30 min of MVPA while at the OSHC service.

**Table 3:** Association of Out of School Hours (OSHC) service physical activity promotion practices on child attainment of more than 30 min of moderate-to-vigorous physical activity (MVPA) in the afterschool period

Physical Activity Promotion Practices	OR	95%CI	*P value*
Physical activity policy			
No (Ref)	-	-	-
Yes	1.0	(0.65, 1.55)	0.968
Staff training in physical activity promotion			
No (Ref)	-	-	-
Yes	1.3	(0.94, 1.96)	0.102
Use of physical activity promotion material			
No (Ref)	-	-	-
Yes	1.0	(0.49, 2.11)	0.953
Recreational screen-time available (TV, movies, computer, video games)			
No (Ref)	-	-	-
Yes	0.8	(0.54, 1.07)	0.121
Recreational handheld devices (i-pad, phones, Tablet) available			
No (Ref)	-	-	-
Yes	0.7	(0.50, 1.02)	0.069
Children’s voices and activity preferences are included in daily programming			
No (Ref)	-	-	-
Yes	1.1	(0.29, 4.00)	0.905
Scheduled time for child-led free play			
≤ 29 min (Ref)	**-**	**-**	**-**
**(30-59 min**)	**2.6**	**(1.70, 3.98)**	** > 0.001**
** ≥ 60 min**	**6.4**	**(3.90, 10.49)**	** > 0.001**
Provision of staff-led organised play			
None (Ref)	-	-	-
(≤ 29 min)	1.4	(0.88, 2.16)	0.157
**(≥ 30 min)**	**2.3**	**(1.47, 3.83)**	** > 0.001**
Active games that engage the majority of children to participate			
No (Ref)	-	-	-
**Yes**	**1.7**	**(1.11, 2.61)**	**0.015**
Active games where children stood still in lines and waited their turn to participate			
No (Ref)	-	-	-
Yes	1.1	0.67, 2.05	0.567
Active games that consist of child elimination components			
No (Ref)	-	-	-
**Yes**	**0.6**	**0.37, 0.86**	**0.008**
Staff engage in physical play with children			
No (Ref)	-	-	-
Yes	0.8	(0.56, 1.04)	0.088

Clustered by OSHC service and child, adjusted for sex and age

Note: Bolded values are significant P < 0.05

## Discussion

This study examined the physical activity (PA) levels and environments in a large sample of Australian OSHC services, operating in the hours after school, and the relationship between physical activity practices and staff behaviours on children’s physical activity levels. We found that children spent an average of 22 min in MVPA, with boys accumulating significantly more MVPA than girls. These findings are similar to international studies conducted in comparable settings, in the United States and Norway [18–21]. Although, sedentary time was much higher within the international literature; a recent meta-analysis exploring physical activity and sedentary behaviours in structured settings, found children attending after school programs (ASPs) spent an average of 54.5 min/d [18] sedentary compared to 36 min/day, within our study. These differences may be attributed to international afterschool programs having longer periods of scheduled sedentary-based activities, including: mandatory cultural studies [19] or allocated homework time [13, 20, 22, 23], compared to Australian OSHC [24]. Although homework and craft-based sedentary activities were offered within our sample, they did not occur at structured time intervals nor were they mandatory.

Across all observation days, 26% of children accumulated 30 min or more of MVPA. Results from the mixed effects logistic regression indicated that the odds of children accumulating 30 min of MVPA increased by 6.4 when services provided 60 min or more of child-led free play and 2.3 times when services scheduled at least 30 min of structured, staff-led organised play into their program. Child-led free play has been recognised throughout the literature as an effective activity type for eliciting high levels of MVPA [20, 22, 25]. However, given the autonomous nature of free-play, it is likely that not all children will choose to engage in active play during this time, therefore organised structured activities may be an important activity to incorporate within the OSHC settings to maximise participation; this could engage a wider range of children, especially girls [26]. The odds of children meeting 30 min of MVPA further increased by 1.7 times when organised activity included games that engaged the majority of children. Given that organised staff-led activities were observed on less than half of observation days and only a quarter of activities included games engaging the majority of children, this identifies a potential area for future intervention within the OSHC setting.

The benefits of structured staff-led organised play within childcare settings, however, have been debated within the broader literature [20, 25, 26], with some studies reporting organised activities to have a lower association with MVPA and higher duration spent idle due to prolonged activity set-up, instructions, and the selection of games that require children to wait their turn or be eliminated from the activity if deemed “out”. This has previously been attributed to a lack of effective staff physical activity  training [21, 27]. It is therefore, not surprising that our results report the odds of children meeting 30 min of MVPA reduced by nearly half when organised games included elimination components to their activities. Unexpectedly, no associations were found between the presence of physical activity policies, staff training or staff engagement in physical activity  with children and an increase in child MVPA. This may be reflective of a lack of specific National/State-level policy or standards surrounding physical activity recommendations within the Australian OSHC setting, and the non-mandatory requirements for service-level physical activity, sedentary behaviour or screen-time policies nor staff physical activity  promotion training. This also may explain the low reported number of physical activity  policies, opportunities for staff physical activity  training, or the limited observed engagement of staff members in physical activity  with children. These results further highlight potential areas for future health promotion opportunities including the development of specific guidelines for the OSHC setting. Additionally, regardless of limited specific guidelines, recreational screen-time was not overtly observed nor was it associated with reduced odds in children meeting 30 min of MVPA. Although recreational screen-time (including handheld devices) was reported to be available (if requested) at 30% of services; it was only observed for more than 30 min on 19% of days, and it was typically available after 16:30 allowing children the opportunity to be physically active for an hour before these devices became available to them.

### Strengths and limitations

This study has several strengths. It is one of the first studies in Australia to use device-based measures of physical activity (accelerometry) to capture child activity levels within OSHC services and explore environmental factors associated with MVPA. Our study used validated observation tools to capture contextual data on physical activity environments and staff behaviours within a large sample of OSHC services. Several limitations must be considered; first, this study was conducted in only two Local Health Districts of NSW, and although this was a large and diverse sample, due to the inconsistency of OSHC requirements between state and territories these findings may not be generalisable outside of NSW. Secondly, there is the potential for staff and children to have modified usual behaviour due to the presence of data collectors. We attempted to reduce this risk by conducting unannounced site visits and asking services to proceed as normal. Furthermore, child activity levels, accumulated during school hours were not captured within this study, as this was beyond the scope of our research.

## Conclusion

OSHC services have the potential to provide positive environments that support physical activity through play and recreation. On average children accrued nearly a quarter of their daily MVPA, with 26% meeting at least 30 min of MVPA while attending OSHC after school. Although this is an encouraging finding, there is the potential for OSHC services to further support children to increase levels of MVPA through play. Results from our study show this could be achieved via scheduling a minimum of 60 min/day for child-led free play, and incorporating opportunities for staff-led organised games for at least 30 min/day, several times per week. When staff lead organised games they should choose activities that engage the majority of children and exclude barriers to physical activity, such as eliminating children from the games. Future interventions should focus on staff training resources or the development of sector-specific physical activity policies/guidelines to assist staff to support children to meet daily physical activity requirements.

## Data Availability

The datasets generated and analysed during the current study are not publicly available as participants did not provide informed consent for data sharing.

## Abbreviations

OSHC: Out of school hours care
NSW: New South Wales
MVPA: Moderate-to-vigorous physical activity
ACECQA: Australian Children’s Education and Care Quality Authority
HAAND: Healthy Afterschool Activity and Nutrition Document
SOSPAN: System for Observing Staff Promotion of Physical Activity and Nutrition
PA: Physical activity
